# More Reliable Neighborhood Contrastive Learning for Novel Class Discovery in Sensor-Based Human Activity Recognition

**DOI:** 10.3390/s23239529

**Published:** 2023-11-30

**Authors:** Mingcong Zhang, Tao Zhu, Mingxing Nie, Zhenyu Liu

**Affiliations:** The School of Computer Science, University of South China, Hengyang 421001, Chinatzhu@usc.edu.cn (T.Z.); lzy@usc.edu.cn (Z.L.)

**Keywords:** human activity recognition, novel class discovery, neighborhood, contrastive learning, similarity, sensor

## Abstract

Human Activity Recognition (HAR) systems have made significant progress in recognizing and classifying human activities using sensor data from a variety of sensors. Nevertheless, they have struggled to automatically discover novel activity classes within massive amounts of unlabeled sensor data without external supervision. This restricts their ability to classify new activities of unlabeled sensor data in real-world deployments where fully supervised settings are not applicable. To address this limitation, this paper presents the Novel Class Discovery (NCD) problem, which aims to classify new class activities of unlabeled sensor data by fully utilizing existing activities of labeled data. To address this problem, we propose a new end-to-end framework called More Reliable Neighborhood Contrastive Learning (MRNCL), which is a variant of the Neighborhood Contrastive Learning (NCL) framework commonly used in visual domain. Compared to NCL, our proposed MRNCL framework is more lightweight and introduces an effective similarity measure that can find more reliable *k*-nearest neighbors of an unlabeled query sample in the embedding space. These neighbors contribute to contrastive learning to facilitate the model. Extensive experiments on three public sensor datasets demonstrate that the proposed model outperforms existing methods in the NCD task in sensor-based HAR, as indicated by the fact that our model performs better in clustering performance of new activity class instances.

## 1. Introduction

With the continuous advancement of deep learning, sensor-based Human Activity Recognition (HAR) has witnessed rapid growth in real-world applications [1,2], such as smart home systems [3], cognitive assistance [4], and health monitoring [5]. These HAR systems typically begin by collecting time-series data generated by various sensors, including accelerometers, gyroscopes, and magnetometers [6]. Subsequently, a large volume of data is manually labeled and used to train a deep learning model, such as CNN [7], DeepConvLSTM [8], and Multi-Head Convolutional Attention [9] and MultiCNN-FilterLSTM [10]. This approach enables the trained model to classify previously labeled activities. However, the aforementioned implementation is limited to operating within a fully supervised setting and does not translate to real-world deployment of HAR systems, in which the activity categories are not defined a priori and massive amounts of sensor data cannot be labeled due to time-intensive and cost-effective constraints [11]. In other words, the primary issue is that HAR systems lack the ability to automatically discover novel activity classes among unlabeled data without external supervision in real-world deployments.

**Figure 1:**
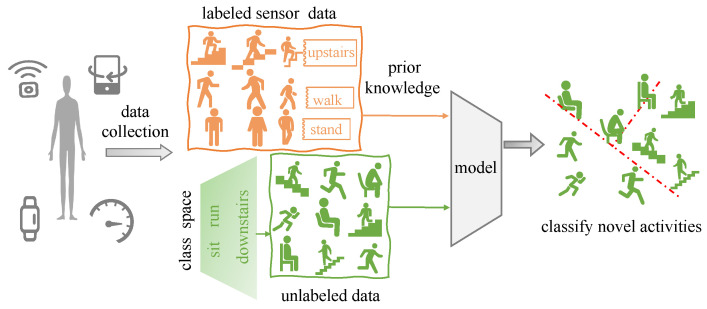
Interpretation of novel class discovery (NCD). The NCD aims to cluster the unlabeled instances into new classes by a model trained through a labeled dataset and an unlabeled dataset that includes disjoint class sets.

In this paper, we investigate the problem of discovering novel activity classes, which we refer to as Novel Class Discovery (NCD). The NCD goal is to learn a model to recognize and classify novel activities. Instead of fully unsupervised settings, we utilize existing labeled data (collected from known class activities) to transfer prior knowledge to unlabeled data that belong to novel class activities completely distinct from the known one. The NCD interpretation is shown in Figure 1.

The NCD problem arises from observations of real-world deployments of HAR. Consider the following scenario: the recognition system continuously collects activity sensor data to train the model. We can manually annotate some activities for model learning. However, hundreds of new activities are introduced every week and providing manual annotations for all activities is hopelessly expensive. The large amount of unlabeled data contains new activities that are not pre-defined and are completely different from the old annotated activity categories. The NCD task is to seek an algorithm that can classify these new activities among massive unlabeled data. In particular, the NCD guides this process by leveraging knowledge from old class activities. The mechanisms of employing transferred knowledge shares a resemblance with the application of transfer learning to HAR. In transfer learning [12,13], given that a labeled source and target dataset share some commonalities, a model is initially trained on the labeled source dataset and subsequently fine-tuned using the labeled information in the target dataset containing distinct activity categories. The fundamental idea behind this approach is to leverage the knowledge of feature representations acquired from the source dataset to enhance the feature learning of the model for the related target dataset. Similar to transfer learning, the NCD case also transfers feature knowledge from a labeled source dataset to a target dataset, whereas no labels are available for the target dataset to fine-tuned model. In other words, our goal is to adapt to the classification task in the target domain without the availability of labels.

In sensor-based HAR, many other areas, such as semi-supervised learning, class incremental learning, and unsupervised clustering, have been working on recognizing activity classes among massive unlabeled data. However, they are relevant but entirely distinct from NCD. Although semi-supervised [14,15] methods employ a small quantity of labeled data to accurately predict activity classes in unlabeled data, the two types of data share the same class space, which is not essentially involved in the discovery of new classes. Class incremental learning [16] continuously recognizes new activity classes along with some tasks, whereas the learning of new classes at each task still requires annotation information. The NCD problem is similar to unsupervised clustering [17,18,19,20,21] that partitions unannotated data into several clusters that are different activity class groups. However, this problem can leverage knowledge of old classes to improve the discovery of the new ones. The NCD task is truly to classify new class activities in unlabeled data by leveraging the labeled data that belong to known activities, and studying this task is actually meaningful for HAR systems in real-world deployments.

**Figure 2:**
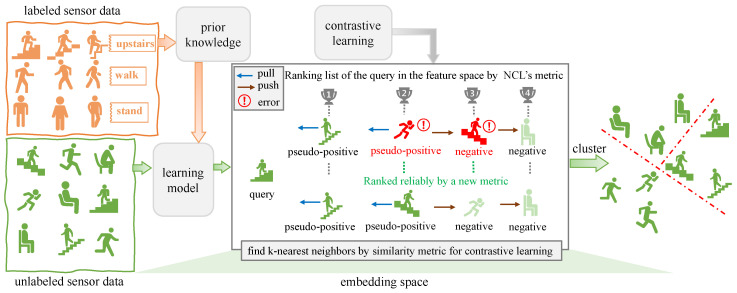
Overall process of More Reliable Neighborhood Contrastive Learning (MRNCL) and advantages of the proposed MRNCL over NCL. Compared with NCL, MRNCL provides more reliable support for similarity ranking between a query sample and other instances in the feature space, ultimately leveraging contrastive learning to make the query closer to its pseudo-positives (neighbors) whereas being away from the negatives.

In NCD task, discriminative inter-class features are beneficial for clustering unlabeled data into new classes. However, as mentioned in [11,22,23], generating distinguishable features to represent activities uniquely is challenging due to the inter-activity similarity [24] with an unsupervised manner. To address this problem, we propose an innovative end-to-end framework, named More Reliable Neighborhood Contrastive Learning (MRNCL). This framework adopts contrastive learning to learn discriminative features from unlabeled data. In NCD scenarios, some labeled data are available, and the MRNCL fully utilizes them to extract prior knowledge to assist in discovering new class activities in unlabeled data. For a given unlabeled query, the MRNCL selects the *k*-nearest neighbors (KNNS) as its pseudo-positives through similarity ranking in the embedding space. The remaining instances not within the KNNs are considered as the query’s negatives. It is likely that these pseudo-positives belong to the same semantic category as the query, unlike the negatives. Subsequently, contrastive learning [25] is applied to bring the query closer to its pseudo-positives while keeping it far away from negatives. Through this mechanism, the model can learn inter-class discrimination and cluster activity instances into several new classes. In fact, our MRNCL is an enhancement and adaptation of the Neighborhood Contrastive Learning (NCL) framework, which is renowned for its application in the NCD task of the visual domain. On the one hand, our MRNCL has reconstructed the NCL framework [26] by removing some components, making our approach more lightweight. On the other hand, in the NCL framework [26], cosine similarly [27] is utilized to select KNNs. It should be noted that the selected KNNs raises a significant issue regarding whether they and their query belong to the same class, which is a crucial factor in the framework’s ability to learn inter-class features and cluster new class instances successfully. As a result, a novel similarity measure is introduced into our MRNCL framework to ensure more reliable neighbors that are most likely to belong to the same semantic category as the query. This measure enhances the performance of our MRNCL relative to NCL in the NCD task of HAR. The overall process of MRNCL and the advantages of this similarity measure over NCL’s [26] are illustrated in Figure 2.

In summary, our contribution is the proposal of an end-to-end framework that automatically classifies novel activity classes within masses of unlabeled sensor data with the assistance of old classes’ activities, addressing the NCD problem that limits the real-world deployments of HAR systems. The framework has two key components. Firstly, contrastive learning is adopted to learn discriminative inter-class features, enabling the model to cluster novel activity instances accurately. Secondly, a novel similarity metric is employed to identify reliable neighbors that are then merged into the contrastive learning process, contributing to the learning of inter-class discrimination. Extensive experimental results on three public sensor datasets demonstrate the practical effectiveness of our proposed model, which outperforms existing methods in the clustering performance of new activity class instances, with significant improvements observed on various datasets.

The remainder of this paper is organized as follows. Section 2 discusses relevant works related to this article. Section 3 describes the MRNCL framework and a new similarity metric for more reliable neighborhoods in detail. Section 4 presents and discusses the results obtained from extensive ablation and comparison experiments, indicating the performance of our proposed framework and new similarity metric. Section 5 discusses the impact of inter-activity similarity on NCD task. In Section 6, the paper is summarized.

## 2. Related Work

### 2.1. Deep Learning for Sensor-Based HAR

In recent years, deep learning has achieved widespread adoption in sensor-based HAR for the real-world implementations of HAR systems [1,2,3,4,5]. Deep learning models, especially convolutional neural networks, recurrent neural networks, and multi self-attention networks, have been very successful in the field of HAR. These models have powerful feature learning and pattern recognition capabilities to automatically extract useful features from raw sensor data. A deep learning model, such as CNN [7], DeepConvLSTM [8], Multi-Head Convolutional Attention [9], and MultiCNN-FilterLSTM [10], is typically trained using a supervised approach on labeled sensor data to enable the recognition and classification of diverse human activities. However, such a supervised model fails to address the open-world challenge [22], as demonstrated by its struggles to recognize novel, unknown activities that were not pre-labeled. This challenge poses a significant obstacle for the practical application of HAR, as providing manual annotations for all novel activities to supervised learning is prohibitively expensive [11]. In this paper, we propose an end-to-end framework to address this challenge by introducing the contrastive learning technique and using a similarity measure component. These two practices empower the deep learning model to automatically discover new class activities even in the absence of sufficient data labels.

### 2.2. Novel Class Discovery

In sensor-based HAR, recognizing novel activities is a significant challenge in the real world scenario [22]. This paper presents an attempt to address this related challenge, referred to as Novel Class Discovery (NCD). In the NCD setting, there exists a labeled dataset that comprises several known class activities, and an unlabeled dataset that comprises novel class activities that are entirely distinct from those in the labeled dataset. The NCD task aims to cluster the data in the unlabeled dataset into the new activity classes by leveraging the existing labeled dataset. Unlike transfer learning [12,13], the transferred knowledge gained from labeled source data is instrumental in enabling the model to learn the feature representation of unlabeled target data, for which no labels are available for model fine-tuning. Unlike class incremental learning [16], NCD learns the new activity classes without any annotated information. Unlike semi-supervised learning [14,15], new activity classes in unlabeled data are completely disjointed with labeled data. Similar to unsupervised clustering [17,18,19,20,21], the NCD aims to classify new activities by clustering unlabeled data into several new classes. However, the NCD differs in its setting to leverage transferred knowledge from labeled data to explore unlabeled datasets. The researches related the NCD problem have made tremendous progress [26,28,29] in computer vision. The proposed MRNCL framework consults the NCL [26] framework to address this problem in sensor-based HAR. Moreover, a valid similarity metric is employed and improves our framework.

### 2.3. Unsupervised Clustering

To our knowledge, unsupervised clustering is most related to our work. It aims to partition a set of unlabeled data into different activity classes without available labeled data. In works [17,18], statistical properties of raw sensor data, including average and standard deviations, are considered as features and then these features are clustered by the *k*-means algorithm [30]. However, high sensitivity to sensor noise [31] are attributed to these methods. The works [19,20] mitigate the noise influence by employing a generative autoencoder (i.e., deep learning model) to generate feature representations of raw data before using *k*-means. However, they have not been able to address the feature space locality limitation [23]. This limitation is reflected that some closely related activities is located closely in the feature embedding space due to the weak feature extraction and ultimately degrades the clustering results. In recent years, hierarchical clustering, such as agglomerative clustering [32] has also emerged in clustering tasks [21], where they analyze the hierarchical relationships between classes to obtain clustering effects. However, they still do not overcome the aforementioned limitation. Instead, this paper proposes the use of a deep learning model to perform end-to-end clustering. In our model, the contrastive learning technique and a similarity measure are used to address weak feature extraction challenges and aggregate class instances.

### 2.4. Contrastive Learning for HAR

Contrastive learning can learn discriminative feature representations between positives and negatives without any label information [25]. In sensor-based HAR, In sensor-based human activity recognition (HAR), its effectiveness for learning discriminative representations has been demonstrated [33,34,35]. In order to cluster new class instances in the NCD task, discriminative inter-class feature extraction is urgently required. The Neighborhood Contrastive Learning (NCL) [26] aims to assign the same-class instances as positive pairs and various-class ones as negative pairs, allowing for the model to learn discriminative inter-class features and effectively cluster unlabeled data. In NCL [26], for an unlabeled query, its *k*-nearest neighborhoods selected by cosine similarity, which are most likely to belong to the same activity class as the query, are considered as its pseudo positives during contrastive learning. This paper proposes a new framework enhanced from the NCL framework and uses an effective similarity to select neighborhoods. Compared to the NCL [26], our MRNCL is lighter, more effective. It is noteworthy that in this paper the contrastive learning technique is also applied to labeled data to assist in exploring unlabeled data.

### 2.5. Similarity Measure Application

The similarity metric is a crucial component in various pattern recognition problems, such as classification [36,37] and clustering [19,20,30,32]. It mainly aims to assist a query in finding its highly similar samples, commonly known as positives. In this paper, the NCL [26] framework utilizes cosine similarity [27] to help a query search for the top-*k* most similar neighbors in the embedding space. These neighbors are expected to belong to the same activity class as the query and subsequently contribute to contrastive learning. However, cosine similarity is unreliable as it only considers directional similarity [38]. Furthermore, the open space risk [39] should be taken into consideration in pattern matching, which suggests that any examples from unknown classes located away from a positive example may be falsely labeled as its “positive”. As stated in the paper [40], the work [36] utilizes multiple similarity measures to process feature representations, reducing the open risk and achieving considerable results. Building on the paper [36], our framework introduces a novel similarity metric by incorporating diverse metrics to perform KNNS, outperforming the original NCL method [26].

## 3. Methods

Problem Formulation. For the NCD task, there is a labeled dataset $D^{l}$ that includes the class set $C^{l}$ and an unlabeled dataset $D^{u}$, including the class set $C^{u}$. Although the two datasets have some degrees of similarity, their corresponding class sets are entirely disjoint. The NCD aims to cluster the $D^{u}$ data into $C^{u}$ based on the knowledge from $D^{l}$.

**Figure 3:**
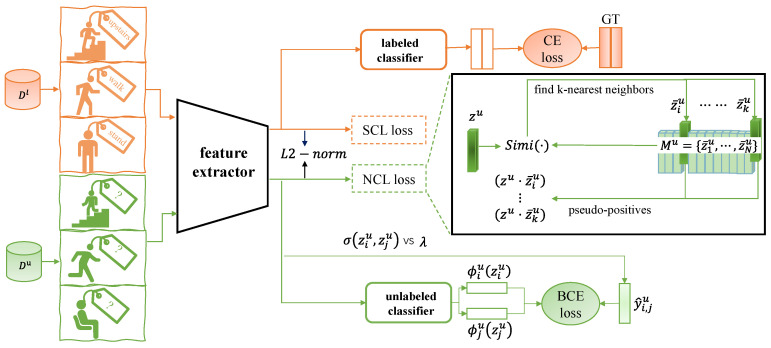
More reliable neighborhood contrastive learning framework for novel class discovery. The feature extractor gains the corresponding representations from the sampled training data, including labeled and unlabeled data. For the labeled data, the CE and SCL losses are calculated through the GT labels. For the unlabeled data, the BCE loss is calculated to optimize the unlabeled classifier and the NCL loss is engaged to learn new-class representations. GT: ground-truth, CE: cross-entropy, BCE: binary cross-entropy, SCL: supervised contrastive learning, NCL: neighborhood contrastive learning, Simi(·): our similarity measure.

Overall Framework. The overall framework of our approach involves a shared feature extractor Ω, which maps input sensor data *x* to a feature vector $z\in R^{H}$. Beneath this feature extractor Ω, there are two classifiers: a labeled linear classifier $\phi^{l}$ and an unlabeled linear classifier $\phi^{u}$, with $C^{l}$ and $C^{u}$ output neurons, respectively. During the model training and learning process, we sample a batch of data from $D^{l}$ and $D^{u}$ and generate two correlated views of the same batch via data augmentation. Then the augmented data are transmitted to Ω. For the labeled samples in $D^{l}$, the extracted feature representations $z^{l}$ are fed into $\phi^{l}$, which is optimized by the cross-entropy (CE) loss through the ground-truth labels. For the unlabeled samples in $D^{u}$, the feature representations $z^{u}$ are generated by the same feature extractor Ω and fed into the classifier $\phi^{u}$. The classifier $\phi^{u}$ then utilizes the binary cross-entropy (BCE) loss to learn to infer the cluster assignments for the unlabeled sensor data. Simultaneously, for the representations $z^{l}$ and $z^{u}$, we redefine them through supervised contrastive learning (SCL) loss and neighborhood contrastive learning (NCL) loss, respectively. By jointly optimizing the CE loss, BCE loss, SCL loss, and NCL loss, our model can effectively learn from both labeled and unlabeled data, resulting in improved clustering performance of unlabeled data. The whole framework is shown in Figure 3.

### 3.1. Baseline Framework

In the labeled dataset $D^{l}=\left\{\left(x_{1}^{l},y_{1}^{l}\right),\cdots ,\left(x_{N}^{l},y_{N}^{l}\right)\right\}$, each sample $x_{i}^{l}$ is explicitly associated with a known class label $y_{i}^{l}$. This label information can be fully leveraged to facilitate the supervised learning of sample features. To achieve this, we optimize the network architecture, which includes the feature extractor Ω and the labeled classifier $\phi^{l}$, using a cross-entropy loss function:

(1) $$l_{\mathrm{ce}}=-\frac{1}{C^{l}}\sum_{i=1}^{C^{l}}y_{i}^{l}log\phi^{l}\left(\Omega \left(x_{i}^{l}\right)\right)$$

In contrast, the unlabeled dataset $D^{u}=\left\{x_{1}^{u},\cdots ,x_{N}^{u}\right\}$ does not come with explicit class labels. Instead, we generate pseudo-labels for pairs of samples $\left(x_{i}^{u},x_{j}^{u}\right)$ in the unlabeled dataset. After feature extraction using Ω, we obtain the feature representations $\left(z_{i}^{u},z_{j}^{u}\right)$ for each pair of samples. Their similarity level is weighted by the cosine similarity [27] $\delta \left(z_{i}^{u},z_{j}^{u}\right)={z_{i}^{u}}^{T}z_{j}^{u}/∥z_{i}^{u}∥∥z_{j}^{u}∥$. When $\delta (z_{i}^{u},z_{j}^{u})$ exceeds a predefined threshold λ, the pairwise pseudo-label is assigned as follows:

(2) $${\hat{y}}_{i,j}=1\left[\delta \left(z_{i}^{u},z_{j}^{u}\right)\geq \lambda \right]$$

where λ represents the minimum similarity required to assign two samples to the same latent class. Then the pairwise pseudo-label is compared to the inner product $p_{i,j}=\phi^{u}{\left(z_{i}^{u}\right)}^{T}\phi^{u}\left(z_{j}^{u}\right)$ obtained from the outputs of unlabeled classifier $\phi^{u}$. The network is optimized by the binary cross-entropy loss:

(3) $$l_{\mathrm{bce}}={\hat{y}}_{i,j}log\left(p_{i,j}\right)+\left(1-{\hat{y}}_{i,j}\right)log\left(1-p_{i,j}\right)$$

In order to make the network generate similar predictions for the sample $x_{i}$ and its correlated view ${\hat{x}}_{i}$, NCL [26] employs mean squared error to calculate consistency loss (CS) for both the labeled and unlabeled samples:

(4) $$\begin{array}{c} l_{\mathrm{mse}}=\frac{1}{C^{l}}\sum_{i=1}^{C^{l}}\left(\phi^{l}\left(z_{i}^{l}\right)-\phi^{l}\left({\hat{z}}_{i}^{l}\right)\right)+\frac{1}{C^{u}}\sum_{j=1}^{C^{u}}\left(\phi^{u}\left(z_{j}^{u}\right)-\phi^{u}\left({\hat{z}}_{j}^{u}\right)\right) \end{array}$$

However, experiments indicate that this methodology is not useful for our model in the NCD task in HAR, so our baseline loss is:

(5) $$l_{\mathrm{base}}=l_{\mathrm{ce}}+l_{\mathrm{bce}}$$

Our baseline loss removes the CS component compared to NCL [26], which is the first reduction.

### 3.2. Supervised and Neighborhood Contrastive Learning

For all the unlabeled samples in a batch, we create two correlated views $\left(x^{u},{\hat{x}}^{u}\right)$ using data augmentation techniques. In traditional contrastive learning, as exemplified in [41], the two views as a positive pair are processed by feature extraction and transformed into $z^{u}$ and ${\hat{z}}^{u}$, respectively. Additionally, a memory bank $M^{u}$ is implemented to store the feature representations of multiple recent batches for training. The elements stored in $M^{u}$ are represented as negative samples ${\bar{z}}^{u}$. And then the contrastive loss for the positive pair is expressed as follows:

(6) $$l_{\left(z^{u},{\hat{z}}^{u}\right)}=-log\frac{e^{\delta (z^{u},{\hat{z}}^{u})/\tau }}{e^{\delta (z^{u},{\hat{z}}^{u})/\tau }+\Sigma_{m=1}^{|M^{u}|}e^{\delta (z^{u},{\bar{z}}_{m}^{u})/\tau }}$$

where δ(·,·) denotes the cosine similarity, and τ is the temperature parameter that controls the scale of distribution.

In (6), the augmented-positive ${\hat{z}}^{u}$ of $z^{u}$ from a given sample is pulled closer whereas negatives ${\bar{z}}_{m}^{u}$ in $M^{u}$ are pushed away from $z^{u}$. This approach ensures that the model learns discrimination for each individual instance. To learn inter-class discrimination, the *k*-nearest neighbor representations that are most likely to belong to the coherent semantic class of $z^{u}$ are selected from $M^{u}$ using a similar measure as shown in the middle of Figure 3, forming a pseudo-positive set:

(7) $$\rho_{k}=\underset{{\bar{z}}_{i}^{u}}{\arg{\mathrm{top}}_{k}}(\{Simi\left(z^{u},{\bar{z}}_{i}^{u}\right)\left|\forall i\in \{1,\cdots ,\right|M^{u}\left|\}\}\right)$$

where Simi(·) is our proposed similar measure, the details of which are provided in Section 3.4. Then the contributions of the examples in $\rho_{k}$ and negatives in $M^{u}$ can be described with the following neighborhood contrastive loss:

(8) $$l_{\mathrm{ncl}}=-\frac{1}{k}\sum_{{\bar{z}}_{i}^{u}\in \rho_{k}}log\frac{e^{\delta (z^{u},{\bar{z}}_{i}^{u})/\tau }}{e^{\delta (z^{u},{\hat{z}}^{u})/\tau }+\Sigma_{m=1}^{|M^{u}|}e^{\delta (z^{u},{\bar{z}}_{m}^{u})/\tau }}$$

The neighborhood contrastive loss in NCL [26] also contains contributions of augment-positives. It is the linear combination of (6) and (8). However, after experimental observations, (6) decreases the accuracy of clustering unknown samples. Thus, our MRNCL removes augment-positives (AP) in neighborhood contrastive loss compared with the NCL [26], which is a two-time reduction.

To acquire robust transferred feature knowledge from labeled data, we also apply the contrastive learning technique to the labeled dataset $D^{l}$. Since the ground-truth labels are available, we can efficiently retrieve the aggregations of positives for a sample $x_{i}^{l}$ with corresponding feature $z_{i}^{l}$ as follows:

(9) $$\rho =\left\{{\bar{z}}_{j}^{l}\in M^{l}:y_{i}^{l}=y_{j}^{l}\right\}\cup {\hat{z}}_{i}^{l}$$

where $M^{l}$ represents the representation queue of the labeled dataset, and ${\hat{z}}_{i}^{l}$ represents the representation of the correlated view. Then, the supervised contrastive loss on labeled data can be defined as follows [42]:

(10) $$l_{\mathrm{scl}}=-\frac{1}{\left|\rho \right|}\sum_{{\overset{˚}{z}}_{j}^{l}\in \rho }log\frac{e^{\delta (z_{i}^{l},{\overset{˚}{z}}_{j}^{l})/\tau }}{e^{\delta (z_{i}^{l},{\hat{z}}_{i}^{l})/\tau }+\Sigma_{m=1}^{|M^{l}|}e^{\delta (z_{i}^{l},{\bar{z}}_{m}^{l})/\tau }}$$

### 3.3. Overall Loss

Compared to NCL [26], our overall loss formulation excludes the consistency loss and the contrastive loss for positive pairs, rendering our model more lightweight. Together with the baseline model, we introduce supervised contrastive learning on labeled data and neighborhood contrastive learning on unlabeled data. The overall loss for our model is:

(11) $$l_{\mathrm{all}}=l_{\mathrm{base}}+l_{\mathrm{scl}}+l_{\mathrm{ncl}}$$

The overall loss is calculated as shown in Algorithm 1.

### 3.4. Similarity Measure for Neighborhoods

Ideally, we would like all the pseudo-positives in $\rho_{k}$ to correspond to the same class as $z^{u}$ enabling our network to learn inter-class discriminative features in a manner similar to supervised contrastive learning. When selecting KNNS as pseudo-positives, we obtain the similarity computation value between $z^{u}$ and ${\bar{z}}_{i}^{u}$ in $M^{u}$ through a similarity metric. The computation value is high, indicating that the ${\bar{z}}_{i}^{u}$ may belong to the same class as $z^{u}$. Then, ${\bar{z}}_{i}^{u}$ corresponding to the top *K* computation values are selected as KNNS of $z^{u}$ (see (7)). The NCL [26] employs cosine similarity to generate $\rho_{k}$, but it is worthwhile to investigate alternative approaches due to its limitations in capturing only directional similarity and the presence of the open space risk [39]. Building on the research of [36], we propose a novel similarity metric to select neighborhoods as pseudo-positives, ensuring that the neighboring instances most likely belong to the same class as $z^{u}$. The following perspectives measure the similarity between $z^{u}$ and ${\bar{z}}_{i}^{u}$ in $M^{u}$.

**Algorithm 1:** Calculation of overall loss.

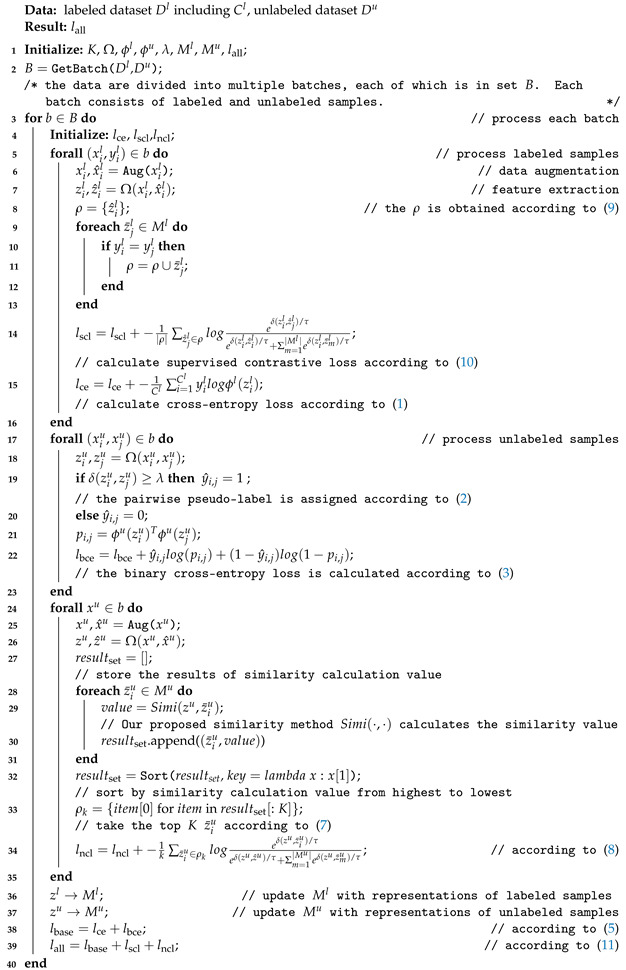



Gower distance [43] is a measurement method that calculates the average of partial differences between instances. It is used to measure the similarity between instances and the Gower distance between $z^{u}$ and ${\bar{z}}_{i}^{u}$ is calculated as follows:

(12) $$d_{\mathrm{gow}}\left(z^{u},{\bar{z}}_{i}^{u}\right)=\frac{1}{n}{∥\frac{z^{u}}{∥z^{u}∥}-\frac{{\bar{z}}_{i}^{u}}{∥{\bar{z}}_{i}^{u}∥}∥}_{1}$$

where *n* represents the dimension of $z^{u}$ (the same as ${\bar{z}}_{i}^{u}$). According to (12), the following similarity metric can be defined:

(13) $$Simi_{\mathrm{gow}}\left(z^{u},{\bar{z}}_{i}^{u}\right)=1-d_{\mathrm{gow}}\left(z^{u},{\bar{z}}_{i}^{u}\right)$$

The Lorentzian distance [44] is a method of measuring the similarity between two sequences. The paper [45], attributed to Lorentzian, introduces a new distance formulation to determine the similarity between instances. The formula for Lorentzian distance is given as follows:

(14) $$d_{\mathrm{Lor}}\left(z^{u},{\bar{z}}_{i}^{u}\right)={∥\ln\left(1+abs\left(z^{u}-{\bar{z}}_{i}^{u}\right)\right)∥}_{1}$$

where abs(·) represents the absolute difference. The addition of 1 ensures the non-negativity property and avoids the occurrence of a logarithm of zero. Based on this distance calculation, the similarity metric $Simi_{\mathrm{Lor}}\left(\cdot \right)$ can be defined as follows:

(15) $$Simi_{\mathrm{Lor}}\left(z^{u},{\bar{z}}_{i}^{u}\right)=1-d_{Lor}\left(z^{u},{\bar{z}}_{i}^{u}\right)$$

Jaccard [46] and Dice coefficients [47] are usually employed to evaluate the similarity between the two sets:

(16) $$\mathrm{Dice}=\frac{2|A⋂B|}{\left|A|+|B\right|}$$

(17) $$\mathrm{Jaccard}=\frac{\left|A⋂B\right|}{\left|A|+|B|-|A⋂B\right|}$$

where *A* and *B* denote the two sets, respectively. Like the research [36], we define the following formula to apply these two schemes to the similarity computation of two instances:

(18) $$Simi_{\mathrm{Dice}}\left(z^{u},{\bar{z}}_{i}^{u}\right)=\frac{2{z^{u}}^{T}{\bar{z}}_{i}^{u}}{∥z^{u}∥+∥{\bar{z}}_{i}^{u}∥}$$

(19) $$Simi_{\mathrm{jac}}\left(z^{u},{\bar{z}}_{i}^{u}\right)=\frac{{z^{u}}^{T}{\bar{z}}_{i}^{u}}{∥z^{u}∥+∥{\bar{z}}_{i}^{u}∥-∥{z^{u}}^{T}{\bar{z}}_{i}^{u}∥}$$

The above multiple similarity measures are combined to obtain our final similarity metric:

(20) $$\begin{array}{ccccc} Simi(z^{u},{\bar{z}}_{i}^{u}) & = & Simi_{\mathrm{gow}}\left(z^{u},{\bar{z}}_{i}^{u}\right)+Simi_{\mathrm{Lor}}\left(z^{u},{\bar{z}}_{i}^{u}\right)\; & + & Simi_{\mathrm{Dice}}\left(z^{u},{\bar{z}}_{i}^{u}\right)+Simi_{\mathrm{jac}}\left(z^{u},{\bar{z}}_{i}^{u}\right) \end{array}$$

Experiments demonstrate that our proposed similarity measure help our framework select more reliable KNNS, thereby improving the clustering accuracy. All over the paper, we refer to a more efficient similarity metric for choosing pseudo-positives in $l_{\mathrm{ncl}}$ collectively as more reliable neighborhood contrastive learning.

## 4. Experiments

### 4.1. Experiment Materials

#### 4.1.1. Dataset

Three publicly available datasets, frequently used in sensor-based HAR researches, are utilized for the experiments on NCD task: WISDM [48], UCI-HAR [49], and USC-HAD [50].

The WISDM dataset [48] comprises data that was gathered using an accelerometer sensor attached to the subjects. The sensor, with three sensor data channels, was set to collect data at a constant frequency of 20 Hz while the 35 subjects engaged in six distinct activities, including downstairs, jogging, sitting, standing, upstairs, and walking. In this study, we employ a sliding window approach to sample the data, utilizing a window length of 90 samples.

The UCI-HAR dataset [49] comprises data records derived from nine sensor channels of the accelerometer and gyroscope sensors. These sensors were sampled at a frequency of 50 Hz. The dataset was collected from 30 subjects, who engaged in six distinct activities, including downstairs, laying, walking, standing, upstairs and sitting. In this study, we sample the data using a sliding window with a length of 128 and a 50% overlap between adjacent windows.

The USC-HAD dataset [50] contains data from 14 people performing 12 activities, including walking-forward, upstairs, walking-left, elevator-down, sitting, elevator-up, walking-right, running, standing, downstairs, jumping, and sleeping. The data were collected from the accelerometer and gyroscope sensors, including six sensor data channels, with a constant rate of 100 Hz. In this study, we sample the data using a sliding window of 200 samples.

In these three processed datasets, all activity instances are labeled, and the activity categories are known. To evaluate the performance of our method on the NCD task, we divide each dataset into a labeled dataset containing known class instance and an unlabeled dataset containing new class instance based on NCD scenarios. For WISDM and UCI-HAR, which, respectively, include six activity categories, the instances belonging to three of the six activity categories are used as the labeled set and the data belonging to the other three are used as the unlabeled set (i.e., the labels are not available). For USC-HAD including twelve activity categories, the labeled and unlabeled sets each contain six activity categories. The goal of NCD aims to cluster the instances of the unlabeled set into corresponding activity categories with the help of the labeled set. The three datasets are partitioned, as listed in Table 1.

#### 4.1.2. Backbone Network

In this paper, our backbone network comprises the DeepConvLstm model [8], serving as a feature extractor, and two dense layers that function as labeled and unlabeled linear classifiers, respectively. The DeepConvLstm model is a classical framework in the field of sensor-based HAR, which encompasses an input layer, four convolutional layers, and two LSTM layers. This framework possesses the ability to automatically learn feature representations and model temporal dependencies between activities. For further details, we refer the reader to the original paper describing the DeepConvLstm model [8].

#### 4.1.3. Implementation Details

For each of three datasets, the training set proportions are all set to 80% and the remaining 20% proportions are as the test set. During the training of the backbone network for NCD task, we use the SGD optimizer update the whole model. The initial learning rate is set to 0.01/0.001 for {WISDM, UCI-HAR}/USC-HAD. For USC-HAD, the learning rate is divided by 10 after 100 epochs. The model is trained with 100/120 epochs in total for {WISDM, UCI-HAR}/USC-HAD. During model training, samples are randomly sampled from both the labeled and unlabeled data, and the batch size is set to 128 for all three datasets. The resampling [34] is employed as the data augmentation method to obtain the two correlated views of instance due to its powerful ability in contrastive learning.

For the binary loss function, λ is set to 0.95. For the NCL loss and SCL loss, they are introduced at the 2th epoch and the temperature parameter τ in them is set to 0.05. We set the queue memory size $|M^{l}\left|=\right|M^{u}|=200/500$ for {WISDM, UCI-HAR}/USC-HAD and the number of pseudo-positives as $K=|M^{u}|/C^{u}/2$. Such parameter settings make our model converge during training and perform well across datasets.

#### 4.1.4. Evaluation Metric

Clustering accuracy is employed to compare the acquired labels with the true labels, which can measure the performance of our model on the NCD task:

(21) $$\mathrm{ACC}=\frac{1}{N}\sum_{i=1}^{N}1\left\{y_{i}^{u}=\mathrm{map}\left({\hat{y}}_{i}^{u}\right)\right\}$$

where $y_{i}^{u}$ and ${\hat{y}}_{i}^{u}$ denote the true and the cluster prediction labels corresponding to $x_{i}^{u}$, respectively. The map(·) indicates the reassignment of the best class label, which can be accomplished by the Hungarian algorithm [51]. In this paper, we primarily utilize the clustering accuracy as our evaluation metric.

To comprehensively assess our method, we employ an additional evaluation metric, the pairwise F-score, in the comparative experiments against other methods. The pairwise F-score measures the clustering quality based on the F-score of all possible pairwise combinations of samples within each cluster. In clustering, the pairwise F-score is calculated for each cluster and then averaged across all clusters to obtain a final score. The pairwise F-score is the harmonic mean of precision and recall, referred to as $F_{P}$ and $F_{R}$, respectively. The formula for pairwise F-score is as follows:

(22) $$F=\frac{2(F_{P}*F_{R})}{F_{P}+F_{R}}$$

where $F_{P}$ is calculated as the number of true positive pairs (i.e., pairs of samples that belong to the same activity and are correctly clustered together) divided by the total number of pairs that are predicted to be positive (i.e., all pairs of samples within the cluster). The $F_{R}$ is calculated as the number of true positive pairs divided by the total number of actual positive pairs (i.e., all pairs of samples in the dataset that belong to the same activity). The formula for $F_{P}$ and $F_{R}$ is as follows:

(23) $$\begin{array}{cc} F_{P} & =\frac{TP}{TP+FP}\\ F_{R} & =\frac{TP}{TP+FN} \end{array}$$

where TP refers to the number of pairs of samples that belong to the same activity and are correctly clustered together. The FP refers to the number of pairs of samples that do not belong to the same activity but are incorrectly clustered together. The FN refers to the number of pairs of samples that belong to the same activity but are not clustered together. A high $F_{P}$ value means that most of the pairs within the cluster are true positive pairs. A high $F_{R}$ value means that most of the true positive pairs in the dataset are included in the cluster. In general, a good clustering result should have both high $F_{P}$ and $F_{R}$ in pairwise F-score.

### 4.2. Ablation Reviews

To evaluate the contributions of each component in MRNCL, we examine different perspectives and present the clustering accuracy (%) on the unlabeled test set in Table 2:

The following considerations can be drawn: In the baseline framework, both CE and BCE play a vital role, and removing each one results in a decrease in the baseline outcomes. On the basis of the baseline, the proposed MRNCL significantly improves the ACC. For example, MRNCL gains +2.81% on WISDM, +1.72% on UCI-HAR, and +1.54% on USC-HAD. Without pseudo-positives, our MRNCL is ineffective on WISDM and even inferior to the baseline on UCI-HAR and USC-HAD. That is because positive feature representations in the memory are treated as negatives and pushed away from the query. The absence of BCE and supervised contrastive learning on labeled data significantly degrades the performance of baseline and MRNCL, respectively. This case illustrates that the prior knowledge learned from the labeled data is beneficial for the NCD task. The above conclusions verify the validity of the MRNCL components.

### 4.3. Reasons for Two Reductions

Compared to NCL [26], the proposed MRNCL discards CS and AP of unlabeled samples. To investigate the impact of these two components, we append them to the model separately for testing. Results of clustering accuracy (%) on the unlabeled test set as presented in Table 3.

As seen in Table 3, appending CS results in a decrease in accuracy on three datasets. CS primarily focuses on enforcing feature consistency between $x_{i}^{u}$ and its correlated view ${\hat{x}}_{i}^{u}$, which only encourages robustness to single-instance variations. However, since we desire the model to learn inter-class variations, CS is not appended to our framework. For appending AP, it is worth noting that there is a catastrophic drop on WISDM and USC-HAD but an improvement on UCI-HAR. This difference may be attributed to the adaptability of the dataset to the data augmentation [34]. The well-adapted UCI-HAR can leverage the augmented-positives to alleviate the influence of the negative samples in the selected KNNS. However, it is challenging to find an augmentation approach that performs well across all sensor datasets in the HAR field [35]. Furthermore, the paper [52] found that augment-positives in contrastive loss (see (6)) classify each activity instance into a single class in the latent space, thereby decreasing the same-class instance aggregations. Therefore, to ensure the generalization of our model, we remove both the consistency loss and augmented-positives.

### 4.4. Comparison with State-of-the-Art Methods

To the best of our knowledge, the NCD task in sensor-based HAR is closely related to unsupervised clustering. Therefore, the proposed MRNCL is compared with commonly used clustering algorithms, such as *k*-means [30] and Agglomerative Clustering (AC) [32], with three different linkage types (i.e., Average, Complete and Ward). For the approach building on *k*-means and AC, a model is first trained using labeled data through supervised learning. Subsequently, features are extracted with such a trained model for the unlabeled data that had never been exploited by the model before. Finally, the *k*-means and AC is applied to these extracted features to acquire the clustering outcomes. Compared with NCL, the proposed MRNCL discards CS and AP, and uses our new similarity measure Simi(·). The modified NCL framework (ModifiedNCL), which removes CS and AP, is chosen for a fair comparison. To show that our framework outperforms the reference framework, we also compare our MRNCL with NCL [26]. The above methods are evaluated on the unlabeled test set by clustering accuracy (%), pairwise F-score (%), precision (%), and recall (%). Comparison results are shown in Table 4.

**Figure 4:**
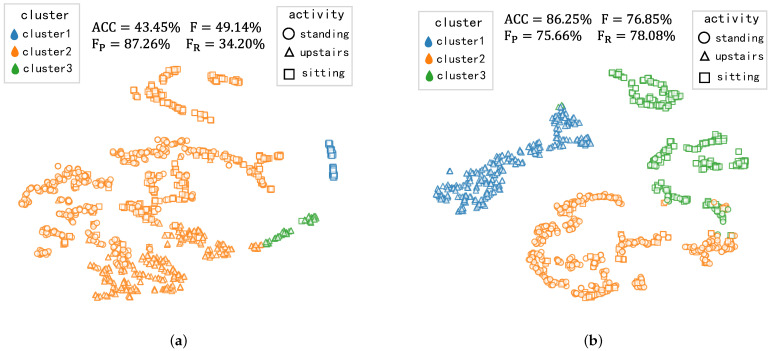
Feature visualization and clustering results on UCI-HAR. The 128-dimensional feature representations (output of the DeepConvLSTM) for unlabeled data are mapped into a 2D embedding space by the t-SNE [53]. We compare our method with *k*-means [30]. Different colors represent different cluster groups, and different point shapes represent the feature representations of different activity samples. (**a**) *k*-means. (**b**) Our MRNCL.

The following observations can be drawn. Firstly, on the WISDN and USC-HAD datasets, the clustering accuracy, pairwise F-score, precision, and recall values are higher than those of *k*-means [30]. On UCI-HAR, our model has a higher recall but a lower precision than *k*-means [30]. In order to gain insights into this phenomenon, we visualize the clustering performance of *k*-means [30] and our method on UCI-HAR. We obtain the feature embeddings of all data form the unlabeled test set on UCI-HAR, which are visualized by t-SNE [53] with dimensionality reduction. Feature visualization and the clustering results by using *k*-means and our method are shown in Figure 4.

As can be observed from Figure 4a, it is evident that cluster1 and cluster3 each correspond to a specific activity(i.e., sitting and upstairs, respectively), which leads *k*-means method to a low false positive rate (i.e., fewer pairs of samples that do not belong to the same activity are incorrectly clustered together). Consequently, the precision rate achieved is relatively high (87.26%). However, a significant number of sitting and upstairs samples are incorrectly clustered in cluster2, which leads to a high false negative rate (i.e., more pairs of samples that belong to the same activity are not clustered together) so that the recall rate only achieves only 34.20%. In contrast, Figure 4b demonstrates that our method yields distinguishable feature representations. This is evident from the observation that different activities are represented in a more compact area. The result of this is that almost all of the upstairs samples are clustered in cluster1, and a majority of sitting samples are clustered in cluster3, which leads our method to enhance the recall rate by +43.88% compared to *k*-means. Furthermore, our clustering accuracy and F-score are significantly higher than *k*-means [30] on UCI-HAR. Taking into account the performance of our method across all three datasets, it is evident that MRNCL is preferred to *k*-means [30] for NCD in HAR.

Secondly, as shown in Table 4, the performance of the three types of linkages in Agglomerative Clustering (AC) is not superior to the *k*-means method on the three datasets. We can observe that on both UCI-HAR and USC-HAD datasets, the AC algorithm with three linkages achieves significantly higher $F_{P}$ but lower $F_{R}$ compared to ours.

Similar to the *k*-means method discussed in the first point, this clustering method is also limited by the feature representations obtained from models trained on labeled data. In other words, the models only capture transferred knowledge of the old classes in the labeled data without performing discriminative feature learning between the new classes for the unlabeled data. This ultimately leads to the fact that the AC algorithm with three different linkages on the three datasets is significantly lower than our MRNCL in terms of ACC and *F*. This once again demonstrates that our model’s ability to learn inter-class features of new activity classes leads to more accurate and superior performance in classifying new activities.

**Figure 5:**
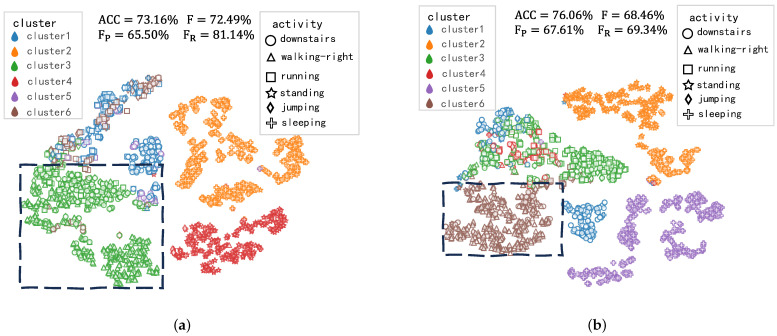
Feature visualization and clustering results on USC-HAD. We compare our method with ModifiedNCL. We also label the two methods on the clustered walking-right activity with black dashed boxes. Different colors represent different cluster groups, and different point shapes represent the feature representations of different activity samples. (**a**) ModifiedNCL. (**b**) Our MRNCL.

Thirdly, as shown in Table 4, our method outperforms ModifiedNCL on WISDN and UCI-HAR datasets, as we achieve higher ACC, *F*, $F_{P}$, $F_{R}$. On USC-HAD, we visualize the feature representations and clustering results of unlabeled samples obtained by these two methods on USC-HAD, as shown in Figure 5.

As shown in Figure 5a,b, it can be observed that neither our method nor ModifiedNCL was able to generate distinguishable feature representations for running and jumping samples. Therefore, the clustering results for these two activity samples are poor under both methods. Due to the inter-activity similarity mentioned in [22,24], similar running and jumping samples cannot be separately classified into their corresponding single cluster, which decreases the recall rate of our method. Inter-activity similarity represents a significant challenge for our work, and its impact on the results is discussed in Section 5. In Figure 5a,b, the feature representations of downstairs, standing, and sleeping samples are all clustered well, as the maximum number of feature representations is represented in a compact area by ModifiedNCL and our method. However, for walking-right samples, ModifiedNCL represents their feature representations in a manner similar to downstairs and running samples, resulting in their incorrect clustering together, as shown in the black dashed box of Figure 5a. In contrast, as shown in the black dashed box of Figure 5b, the feature representations belonging to walking-right samples are easily distinguishable from those of other activities Therefore, they are grouped into a more compact area and well clustered together, leading to a +2.9% increase in ACC and a +2.11% increase in $F_{P}$ for our method compared to ModifiedNCL. The above performance on three datasets demonstrates that our similarity metric facilitates the model in learning inter-class features between new classes, ultimately improving the clustering results.

Fourthly, in contrast to NCL [26], our lighter MRNCL still boosts ACC by +9.52% on WISDM, +1.34% on UCI-HAR, and +1.12% on USC-HAD as shown in Table 4. Additionally, our method outperforms the NCL framework in terms of *F*, $F_{P}$, $F_{R}$. Therefore, our MRNCL is superior to the NCL framework.

Overall, the performance of our framework on the three datasets demonstrates the strong capability of our method to effectively classify unlabeled data into distinct new activity categories on the new class discovery task in sensor-based HAR.

### 4.5. More Reliable Neighborhoods

The main advantage of our MRNCL over NCL [26] lines in our similarity measure, which helps to select more reliable neighborhoods as pseudo-positives for contrastive loss.

This enables our model to learn inter-class features for clustering new class samples and improving clustering results. To demonstrate the reliability of our proposed similarity metric in selecting neighborhoods, we calculate the percentage (%) of true positives (i.e., they and their query are the same activity class) in the selected KNNS for each epoch during training steps, and then take the average of all calculated values across both three datasets.

Our approach is compared to the following three approaches.

1. Cosine during ModifiedNCL: the ModifiedNCL framework, employing the cosine similarity to select neighborhoods. Compared with NCL [26], this framework removes CS and AP.
2. Cosine during NCL: the original NCL framework [26], employing the cosine similarity for neighborhoods.
3. Simi during MRNCL: our framework, which employs a new similarity measure, Simi(·).

**Figure 6:**
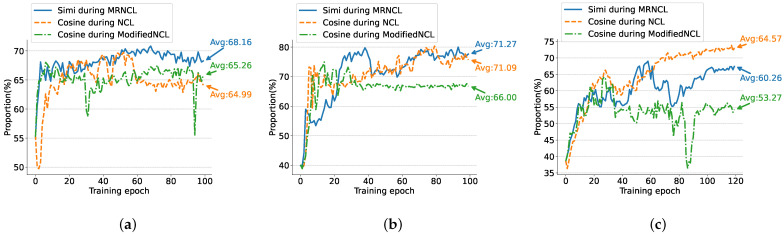
The proportion of true-positives for contrastive loss in elected KNNS during training on three datasets: (**a**) WISDN, (**b**) UCI-HAR, and (**c**) USC-HAD. Avg: The average of all epoch calculated values.

The results are displayed in Figure 6.

The following conclusions can be drawn from Figure 6. Firstly, when comparing our MRNCL with ModifiedNCL, the similarity metric is the only difference between the two frameworks. Our Simi(·) selects significantly more true positives than the cosine similarity as the training epochs increase on all three datasets. This is evidenced by the fact that our method has an average increase of +2.9% on WISDM, +5.27% on UCI-HAR, and +6.99% on USC-HAD. More true positives reinforces our model’s ability to learn inter-class features of new classes, thereby improving the clustering results, as shown in Table 4. Additionally, as shown in Figure 6a,c, the cosine similarity in ModifiedNCL leads to significant instability in the selection of true positives during training. Therefore, our similarity measure is significantly superior to the cosine similarity for finding more reliable neighborhoods. Secondly, NCL [26], which employs the CS and AP forces, outperforms ModifiedNCL when using the cosine similarity for true positives on UCI-HAR and USC-HAD. However, as displayed in Figure 6a, NCL [26] becomes unreliable after 60 epochs of training on the WISDM dataset, reducing the ACC by 5.9%, *F* by 6.24% as shown in Table 4. Finally, compared with NCL [26], our framework still performs Avg +3.17% on WISDM and +0.18% on UCI-HAR even without CS and AP. This further demonstrates the superiority of our similarity metric. On USC-HAD, although the Avg of true positives is higher than ours, the clustering performance is inferior to ours, as shown in Table 4, This indicates the reliability of our lighter framework.

**Figure 7:**
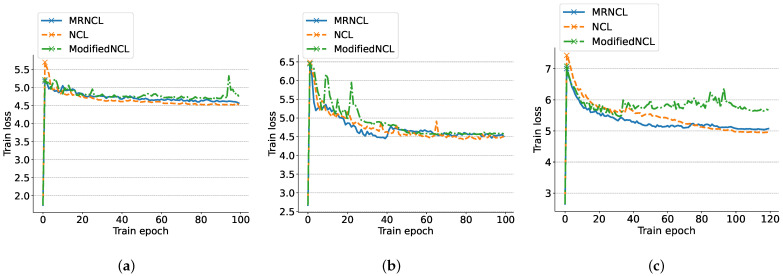
Training loss vs. epochs plots for the three models on the three datasets: (**a**) WISDN, (**b**) UCI-HAR, and (**c**) USC-HAD.

To demonstrate the robustness of the model during training, we also present the correlation between the number of epochs and loss for the three models on the three datasets in Figure 7.

In Figure 7, we observe a significant increase in the training loss value at the beginning of the epoch for all three datasets across all three frameworks. This increase is attributed to the introduction of the NCL loss and SCL loss at the 2th epoch, as discussed in Section 4.1.3. As can be seen from Figure 7a,c, the ModifiedNCL experiences fluctuations in the loss value between 80 and 100 epochs during training on WISDN and USC-HAD. This instability is attributed to the cosine similarity’s inconsistent ability to select true-positives as neighborhoods (presented in Figure 6a,c), which impacts the model’s stability. In contrast, the loss value decreases gently and converges at the end of training for our MRNCL, highlighting its exceptional robustness and reiterating the superiority of our similarity metric. Figure 7b demonstrates that both MRNCL and NCL exhibit consistent and gradual loss convergence, with a slight increase in the middle, indicating their effectiveness in learning and optimizing the given task. Across all three datasets, our MRNCL and NCL exhibits a smooth performance during training. However, our MRNCL has neither CS nor AP components, indicating its lightweight nature compared to NCL.

## 5. Discussion

**Figure 8:**
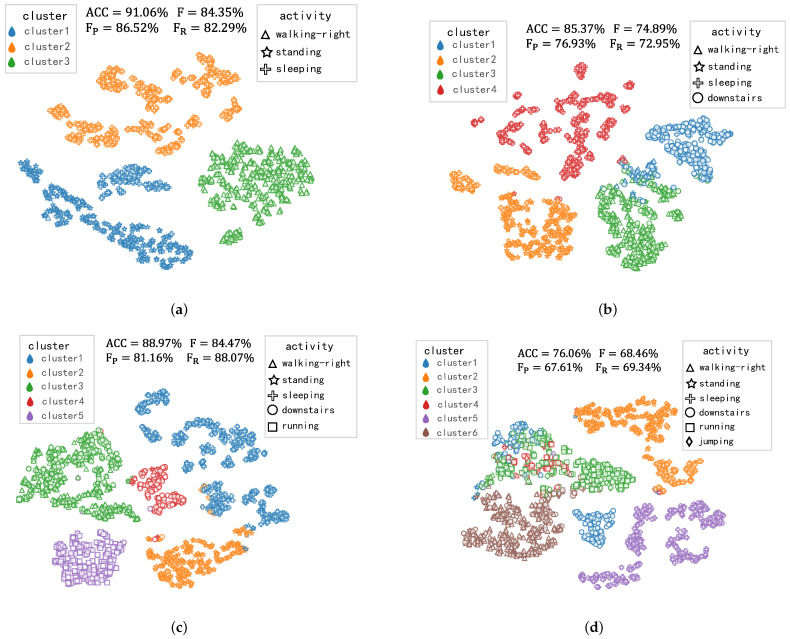
The clustering visualization results of different unknown activities in the unlabeled dataset on USC-HAD. Different colors represent different cluster groups, and different point shapes represent the feature representations of different activity samples. (**a**) Initially three activity categories; (**b**) initially three activity categories, downstairs; (**c**) initially three activity categories, downstairs and running; and (**d**) initially three activity categories, downstairs, running, and jumping.

In this study, we have presented a theoretical and experimental exploration of the NCD process in sensor-based HAR. Across three publicly datasets, our framework exhibited superior performance in the task of NCD. However, the similarity between different activities can significantly impact the classification of new class activities in unlabeled data. To investigate the influence of inter-activity similarity on NCD task, we conduct a comparative study on the USC-HAD dataset. Throughout the experiment, the activity categories in the labeled dataset remain unchanged, as presented in Table 1. For the unlabeled dataset, we initially include three easily distinguishable activities: walking-right, standing, and sleeping. Subsequently, downstairs, running, and sleeping activities are sequentially added to the unlabeled dataset. The clustering visualization results of different unknown activities are illustrated in Figure 8.

As shown in Figure 8a, walking-right, standing, and sleeping activities exhibit significant dissimilarity, making it relatively easy to classify them. This results in a clustering accuracy of 91.06% and a pairwise F-score of 84.35%. In Figure 8b, the downstairs and walking-right activities are highly similar, leading to misclassification and drop in accuracy to 85.37% and F-score of 74.89%. Compared to Figure 8b, Figure 8c adds the running activity category but the clustering accuracy and pairwise F-score improve to 88.97% and 84.47%, respectively. In Figure 8c, the running activity is clearly separated from the other activities while walking-right remains confused with downstairs activity. From Figure 8b,c, it indicates that the number of new activity categories does not significantly impact the clustering performance of the new class activities. In Figure 8d, running and jumping activities are difficult to classify due to their high inter-class similarity. Therefore, it is crucial to consider the influence of inter-activity similarity [24] on both recognition accuracy and the ability to discover new activities in our framework for the NCD task.

## 6. Conclusions

This paper presents the attempt to study the new class discovery problem in sensor-based Human Activity Recognition. A holistic framework called More Reliable Neighborhood Contrastive Learning (MRNCL) is proposed to address this problem. This framework references a visual domain framework but is more lightweight and effective than the reference framework. In the proposed framework, we employ a new similarity measure to select more reliable neighborhoods for unlabeled queries in the embedding space and adopt contrastive learning to take the knowledge from these neighborhoods to improve the clustering accuracy. Experiments on three datasets demonstrate that our method is effective in sensor-based HAR for new class discovery task. We hope that our work will kindle further research in this crucial direction.

## Figures and Tables

**Table 1:** Dataset statistics for MRNCL.

Dataset	Labeled Set			Unlabeled Set		
	Instance	Class	Activity	Instance	Class	Activity
WISDM	≈5 K	3	downstairs, jogging, sitting	≈5.2 K	3	standing, upstairs, walking
UCI-HAR	≈6.3 K	3	downstairs, laying, walking	≈7.4 K	3	standing, upstairs, sitting
USC-HAD	≈12 K	6	walking-forward, upstairs, walking-left	≈12 K	6	walking-right, running, standing
			elevator-down, sitting, elevator-up			downstairs, jumping, sleeping

**Table 2:** Ablation study of the proposed MRNCL. **Baseline**: our baseline framework; **Basel. w/o CE**: the cross-entropy (CE) loss is removed from the baseline; **Basel. w/o BCE**: the binary cross-entropy (BCE) loss is removed from the baseline; **MRNCL**: our proposed MRNCL; **MRNCL w/o PP**: no neighborhoods are used as pseudo-positives for the contrastive loss; **MRNCL w/o LA**: no supervised contrastive learning on labeled data.

Method	WISDM	UCI-HAR	USC-HAD
**Basel. w/o CE**	70.17	35.91	49.05
**Basel. w/o BCE**	71.85	55.21	57.37
Baseline	80.97	84.53	74.52
+**MRNCL w/o PP**	81.77 (↑0.8)	83.86 (↓0.67)	73.28 (↓1.24)
+**MRNCL w/o LA**	83.71 (↑2.74)	81.57 (↓2.96)	60.31 (↓14.21)
+**MRNCL**	**83.78 (↑2.81)**	**86.25 (↑1.72)**	**76.06 (↑1.54)**

**Table 3:** Influence of the consistency loss and augment-positives. **MRNCL w CS**: append consistency loss to our framework, **MRNCL w AP**: append augment-positives.

Method	WISDM	UCI-HAR	USC-HAD
**MRNCL**	**83.78**	86.25	**76.06**
**MRNCL w CS**	82.31 (↓1.47)	85.29 (↓0.96)	65.95 (↓10.11)
**MRNCL w AP**	71.78 (↓12)	**88.16 (↑1.91)**	71.79 (↓4.27)

**Table 4:** Comparison with state-of-the-art methods on three datasets for novel class discovery.

Method	WISDM				UCI-HAR				USC-HAD			
	ACC	F	$F_{P}$	$F_{R}$	ACC	F	$F_{P}$	$F_{R}$	ACC	F	$F_{P}$	$F_{R}$
*k*-means	69.97	61.94	54.91	71.03	43.45	49.14	87.26	34.20	72.07	65.02	64.70	65.34
AC-Average	68.70	61.12	54.19	70.09	42.44	49.11	88.58	33.97	66.90	61.27	**80.99**	49.28
AC-Complete	66.82	59.65	52.67	68.77	42.79	49.13	88.11	34.06	63.05	60.11	72.80	51.19
AC-Ward	68.23	60.94	55.50	67.57	42.22	49.14	**88.89**	33.95	65.91	60.93	66.68	56.10
ModifiedNCL	80.16	76.02	71.08	81.70	83.48	72.75	70.35	75.31	73.16	**72.49**	65.50	**81.14**
NCL	74.26	69.78	68.52	71.09	84.91	75.18	73.11	77.38	74.94	67.75	67.40	68.10
MRNCL(ours)	**83.78**	**80.82**	**71.70**	**92.60**	**86.25**	**76.85**	75.66	**78.08**	**76.06**	68.46	67.61	69.34

## Data Availability

Publicly available datasets were analyzed in this study.

## Abbreviations

The following abbreviations are used in this manuscript:

HAR	Human Activity Recognition
NCD	Novel Class Discovery
NCL	Neighborhood Contrastive Learning
KNNS	*K*-nearest neighbors
MRNCL	More Reliable Neighborhood Contrastive Learning
GT	Ground-truth
CE	Cross-entropy
BCE	Binary cross-entropy
SCL	Supervised contrastive learning
CS	Consistency loss
AP	Augment-positives
AC	Agglomerative Clustering
